# Comparison of the Illumina NextSeq 2000 and GeneMind Genolab M sequencing platforms for spatial transcriptomics

**DOI:** 10.1186/s12864-023-09192-w

**Published:** 2023-03-07

**Authors:** Iamshchikov Pavel, Larionova Irina, Gerashchenko Tatiana, Piankov Denis, Koshkin Philipp, Korostelev Sergei, Denisov Evgeny

**Affiliations:** 1 Laboratory of Cancer Progression Biology, Cancer Research Institute, Tomsk National Research Medical Center, Russian Academy of Sciences, Tomsk, 634009 Russia; 2 Laboratory of Translational Cellular and Molecular Biomedicine, Tomsk State University, Tomsk, 634050 Russia; 3 Laboratory of Single Cell Biology, Research Institute of Molecular and Cellular Medicine, Peoples’ Friendship University of Russia (RUDN University), Moscow, 117198 Russia; 4 Laboratory of Molecular Pathology, Genomed Ltd, Moscow, 115093 Russia

**Keywords:** GeneMind Genolab M, Illumina NextSeq 2000, 10x Genomics Visium, Sequencing, Spatial transcriptomics

## Abstract

**Background:**

The Illumina sequencing systems demonstrate high efficiency and power and remain the most popular platforms. Platforms with similar throughput and quality profiles but lower costs are under intensive development. In this study, we compared two platforms Illumina NextSeq 2000 and GeneMind Genolab M for 10x Genomics Visium spatial transcriptomics.

**Results:**

The performed comparison demonstrates that GeneMind Genolab M sequencing platform produces highly consistent with Illumina NextSeq 2000 sequencing results. Both platforms have similar performance in terms of sequencing quality and detection of UMI, spatial barcode, and probe sequence. Raw read mapping and following read counting produced highly comparable results that is confirmed by quality control metrics and strong correlation between expression profiles in the same tissue spots. Downstream analysis including dimension reduction and clustering demonstrated similar results, and differential gene expression analysis predominantly detected the same genes for both platforms.

**Conclusions:**

GeneMind Genolab M instrument is similar to Illumina sequencing efficacy and is suitable for 10x Genomics Visium spatial transcriptomics.

**Supplementary Information:**

The online version contains supplementary material available at 10.1186/s12864-023-09192-w.

## Background

Single-cell RNA sequencing (scRNA-seq) has become a powerful approach to characterize the gene expression profile in single cells [1]. This method allows processing tens and hundreds of thousands of single cells simultaneously to measure their transcriptional profiles [2, 3]. ScRNA-seq technique provides a higher resolution of cellular differences and better understanding of cell populations and their relationships [4, 5]. However, a serious practical obstacles related to cell stress, cell death, and/or cell aggregation during cell isolation for scRNA-seq exist, accompanied by the loss of spatial context. Moreover, some cell types in tissue, in particular immune cells, are not always easily isolated from tissue [6].

Spatial transcriptomics provides quantitative gene expression data within the spatial context of tissues and cells on tissue sections [7]. Among single-cell spatial transcriptomics methods, 10x Genomics Visium is a barcoding technique that gives an information about cell-type composition on the spots [8]. While “bulk” cDNA libraries have relatively plain structure, Visium cDNA libraries are composed of several elements making these libraries more sophisticated. For example, Visium libraries obtained from formalin-fixed paraffin-embedded (FFPE) samples include spatial barcodes, unique molecular identifiers (UMI) at the Read 1, and probe sequences at the Read 2. Base calling errors at these library elements could cause loss of information about spatial position, UMI assignment, and probe origin. Therefore, these complicated libraries demand high sequencing quality.

The Illumina sequencing systems demonstrate high efficiency and power and remain the most popular platforms that is confirmed by the dominance in the literature and in the amount of data submitted to the Sequence Read Archive [9]. Platforms with similar throughput and quality profiles but lower costs (cost per read) are under intensive development [10].

In October 2020, GeneMind Biosciences Company Limited (GeneMind) launched a new sequencing instrument (GenoLab M) that is fully compatible with Illumina based libraries and does not require any library conversion [11]. It was revealed that the GenoLab M is a promising sequencing platform for transcriptomics and long non-coding RNA profiling in animal, plant, and human with comparable performance but a lower cost compared to NovaSeq 6000 (Illumina) [11, 12]. However, the performance of the GenoLab M platform in other applications has not yet been tested, for example, for single cell sequencing including spatial transcriptomics.

Here, we compared the relative performance of GeneMind Genolab M and Illumina NextSeq 2000 for 10x Genomics Visium spatial sequencing. The same cDNA libraries prepared from three ovarian cancer samples were sequenced in two platforms, and the efficacy and accuracy of results were analyzed by bioinformatics.

## Results

### Quality of sequencing

In order to assess concordance of the Illumina NextSeq 2000 and GeneMind Genolab M sequencing platforms for spatial transcriptomics, we sequenced three 10x Genomics Visium libraries of FFPE ovarian tumor tissues. The main metrics are provided in Table 1.

**Table 1:** Unity of sequencing quality of the samples in two sequencing platforms

Slide	A1-1		A1-2		B1-2	
Platform	NextSeq 2000	Genolab M	NextSeq 2000	Genolab M	NextSeq 2000	Genolab M
Number of Reads	57,183,864	53,580,387	70,380,616	64,184,316	88,306,199	68,468,084
Valid Barcodes	98.20%	97.60%	97.40%	96.70%	98.30%	97.40%
Valid UMIs	100.00%	99.90%	100.00%	99.90%	100.00%	99.90%
Sequencing Saturation	31.90%	10.70%	69.70%	62.90%	48.80%	29.00%
Q30 Bases in Barcode	96.50%	95.50%	96.70%	94.70%	96.60%	94.90%
Q30 Bases in Probe Read	97.00%	95.10%	96.70%	94.30%	97.20%	94.80%
Q30 Bases in UMI	97.20%	95.20%	97.40%	94.40%	97.30%	94.60%

The mean number of reads for the NextSeq 2000 and Genolab M platforms was about 72 and 62 million, respectively. The percentage of valid barcodes and percentage of valid UMIs were 0.74% and 0.1% higher in NextSeq 2000. Base calling accuracy was also higher in the NextSeq 2000. The percentage of Q30 bases (99.9% base call accuracy) in barcode, probe, and UMI reads was about 1.6%, 2.2%, and 2.6% higher in the NextSeq 2000, respectively. Furthermore, there was apparent difference in the sequencing saturation, which was higher when using NextSeq 2000. This could be due to the fact that the sequencing depth and the number of read duplication were lower with using the Genolab M platform (Supplementary Table 1).

### Raw reads mapping and counting

The raw sequencing reads from two sequencers were aligned to the probe reference and assigned to tissue spots by corresponding barcode sequences, and then the aligned and assigned reads were counted. The summarized quality metrics of processed data are included in Table 2. The fraction of reads under tissue spots was consistent between two platforms with insignificant differences. The mean number of reads per spot and the mean number of reads per spot under tissue were in accordance with the sequencing depth. The median number of UMI counts and genes per spot and the number of detected genes were higher in the Genolab M platform, excepting the number of detected genes in B1-2 slide. However, uniquely detected genes in both platforms had markedly low number of UMIs (Supplementary Figure S1A, B) suggesting that these genes have sporadic origin. The percentage of confidently mapped reads to probe set was slightly higher for NextSeq 2000 that is concordant with higher sequencing quality of the probe reads. Nevertheless, Genolab M showed the higher percentage of confidently mapped reads to filtered probe set.

**Table 2:** Quality control metrics of the raw read mapping and counting

Slide	A1-1		A1-2		B1-2	
Platform	NextSeq 2000	Genolab M	NextSeq 2000	Genolab M	NextSeq 2000	Genolab M
Fraction Reads in Spots Under Tissue	80.30%	80.40%	83.30%	83.40%	70.30%	70.20%
Mean Reads per Spot	20,278	19,000	27,482	25,062	44,622	34,597
Mean Reads Under Tissue per Spot	15,975	14,885	15,672	14,174	25,492	19,558
Median UMI Counts per Spot	10,744	12,618	2,751	2,918	10,275	10,405
Median Genes per Spot	4,565	5,034	1,647	1,736	3,866	3,930
Genes Detected	16,736	16,852	16,441	16,532	16,779	16,767
Reads Mapped to Probe Set	98.60%	98.30%	96.10%	95.90%	98.10%	97.50%
Reads Mapped Confidently to Probe Set	97.50%	97.30%	60.80%	60.30%	70.90%	70.50%
Reads Mapped Confidently to the Filtered Probe Set	73.30%	74.20%	44.80%	45.50%	55.10%	55.50%

The filtered probe set is the probe set filtered from hybridization probes with high similarity to potential non-specific target genes, so reads from this set are mapped to unique genes. However, the differences between read mapping to the filtered probe set were insubstantial and could arise sporadically.

### Characterization of the genes, UMIs, reads, and tissue spots

**Fig. 1:**
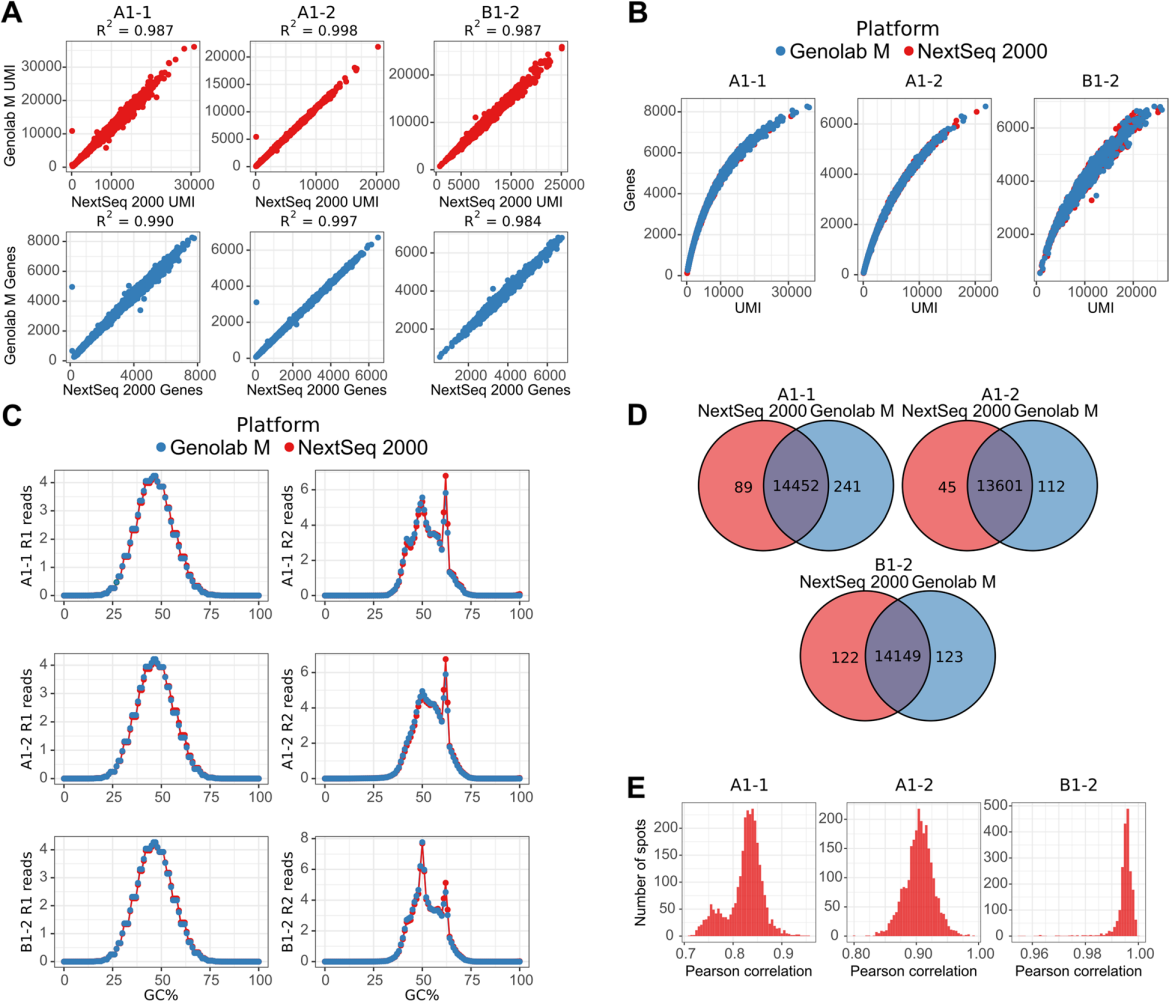
Characterization of the read counts, UMIs, and detected genes obtained from two sequencing platforms. **A** Correlation between the number of transcripts (UMIs) and the number of detected genes in the tissue spots. **B** The genes and UMIs distribution in the tissue spots in two sequencing platforms. **C** GC-content profiles of raw reads in two sequencing platforms. **D** The unique and common genes between two sequencing platforms. **E** Distribution of the Pearson correlation coefficients of the common SCT transformed gene counts

The raw sequencing reads were converted into the feature-barcode matrix following a read mapping and counting in the Space Ranger. The resulting feature-barcode matrixes were loaded into the R environment for downstream analysis in the Seurat R package. The Pearson correlation coefficient was used to assess concordance of the sequencing results in the tissue spots. There were strong correlations between the total number of UMIs and the total number of detected genes in the tissue spots (Fig. 1A). The gene-UMI relationship (Fig. 1B) for all samples was consistent between two platforms and in accordance to expected distribution. We surveyed GC-content profiles in both forward and reverse reads. GC-content profiles were highly consistent between two platforms (Fig. 1C), indicating no any apparent biases between read coverage in two sequencers.

In order to perform downstream analysis, we filtered out low expressed genes and tissue spots with insufficient number of genes using the following thresholds: genes expressed in less than 10 tissue spots and tissue spots with less than 200 filtered genes. Then, overlapping of the filtered genes between two platforms was examined (Fig. 1D). Since there is no well-established criterion to filter out low-expressed genes and tissue spots, different studies choose different thresholds and criterions. The main purpose of filtering is removing low expressed genes, which can affect dimension reduction processes introducing additional noise to data. The thresholds in our manuscript were chosen by survey articles where 10x Genomics Visium technology was used. Closest thresholds to our analysis were used within the article [13].

The number of unique genes in the Genolab M platform was still higher than in the NextSeq 2000, while the proportion between unique and overlapping genes after filtration in both platforms became lower.

Nevertheless, more detailed insight revealed that all unique genes in both platforms had poor coverage and distributed on the edge of the chosen threshold for gene filtration (Supplementary Figure S1C) that indicate their sporadic nature. The read counts were normalized via the SCTransform function in the Seurat package. The SCTransform utilized regularized negative-binomial regression to remove the influence of sequencing depth and other specified unwanted sources of variation. The normalized counts were used for correlation analysis between the overlapping genes (Fig. 1D). There were strong correlations between overlapping genes in spots sequenced on both platforms. Mean correlation coefficients were 0.82, 0.90, and 0.99 in A1-1, A1-2, and B1-2 samples, respectively (Fig. 1E). Analysis of the sequencing quality metrics did not reveal any robust metrics explaining differences in correlation coefficients obtained for the samples.

### Dimension reduction, clusterization, and differential expression

**Fig. 2:**
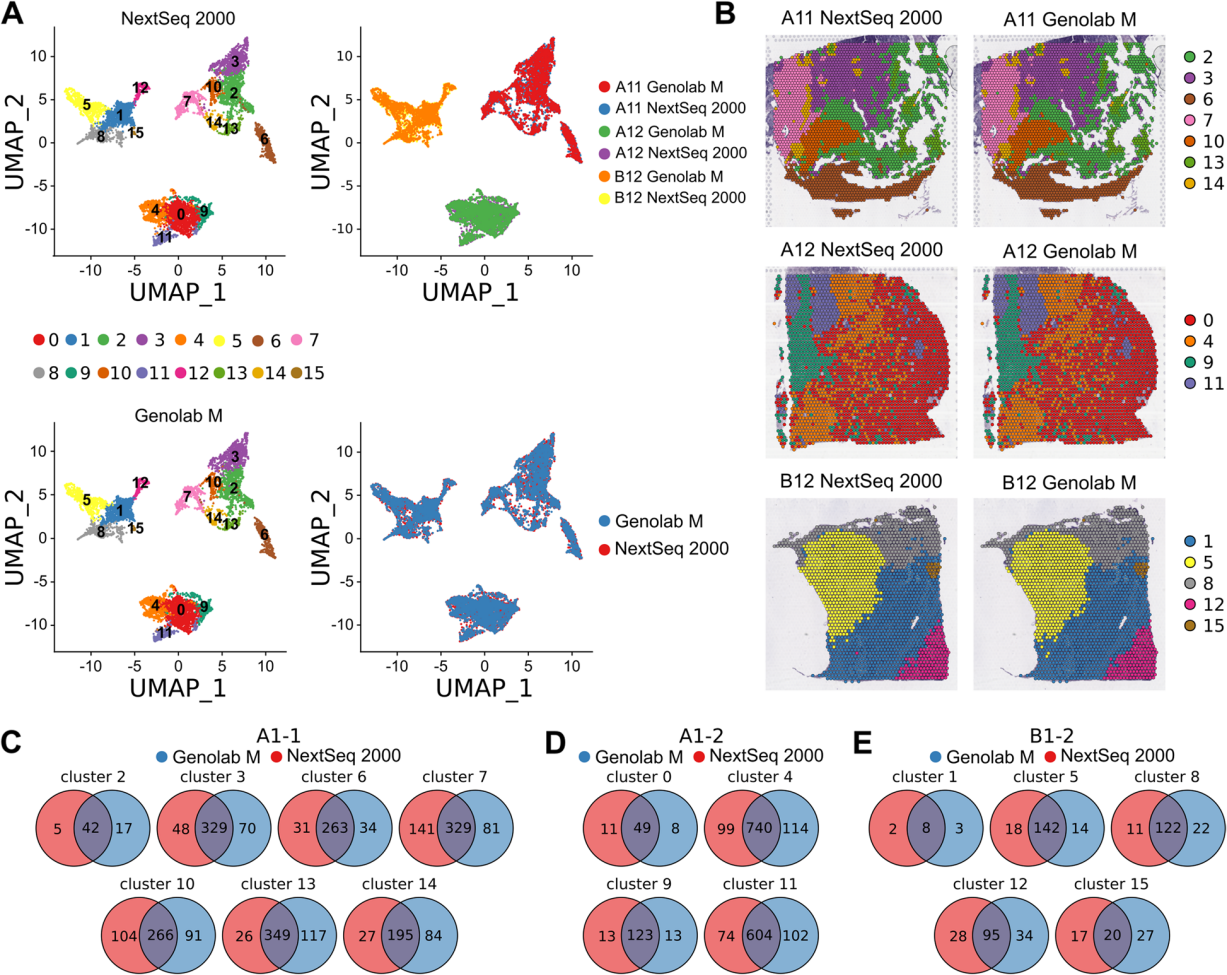
Downstream non-linear dimension reduction, clusterization, and differential expression of the filtered and normalized data. **A** The UMAP dimension reduction and clusterization results of the sequencing counts from two sequencing platforms without batch effect correction. **B** Spatial distribution of the obtained clusters. **C-E** Venn diagrams of the common and the platform-unique differentially expressed genes (DEGs) of the slide clusters

The normalized read counts were merged and renormalized via the SCTransform function. Figure 2A demonstrates no observable batch effect between samples sequenced on NextSeq 2000 and Genolab M. In further analysis, SCTransform function without batch effect correction was applied. The resulted SCT normalized counts were used for linear dimension reduction via PCA approach, and the first 30 principal components were used for clusterization and non-linear dimension reduction. SNN analysis based clustering algorithm was used for clusterization of tissue spots. UMAP approach was used for visualization of tissue spots and clusterization in two dimensions. Results of SNN clusterization and UMAP dimension reduction are depicted in Fig. 2A. Tissue spots from the samples analyzed by different sequencing platforms were placed in close proximity. Figure 2B demonstrates cluster distribution on the tissue sections. There were not recognizable differences between spatial cluster distribution in spots sequenced on both platforms. A1-1 sample had the most complex structure regarding number of defined clusters and their distribution on the tissue section. A1-2 sample had less number of clusters, and B1-2 section had simplest spatial distribution of clusters. Collectively, aforementioned evidence regarding spatial clustering could account for difference in tissue spot correlation coefficients between three samples.

Since there is no observable batch effect, DEG analysis can be performed directly on counts after sequencing depth normalization via SCTransform. The SCT normalized counts were used in the Wilcoxon Rank Sum test to find differentially expressed genes in each sample separately. Genes were considered differentially expressed if FDR < 0.01 and LFC > 0.25. Figure 2C-E demonstrates results of differential expression analysis. The number of unique genes detected by NextSeq 2000 and Genolab M is around 16% out of all DEGs detected by both platforms for all clusters; the overlapping rate is about 68%. Therefore, DEGs predominantly overlapped between same clusters originated from two sequencing platforms.

### Characterization of differentially expressed genes

**Fig. 3:**
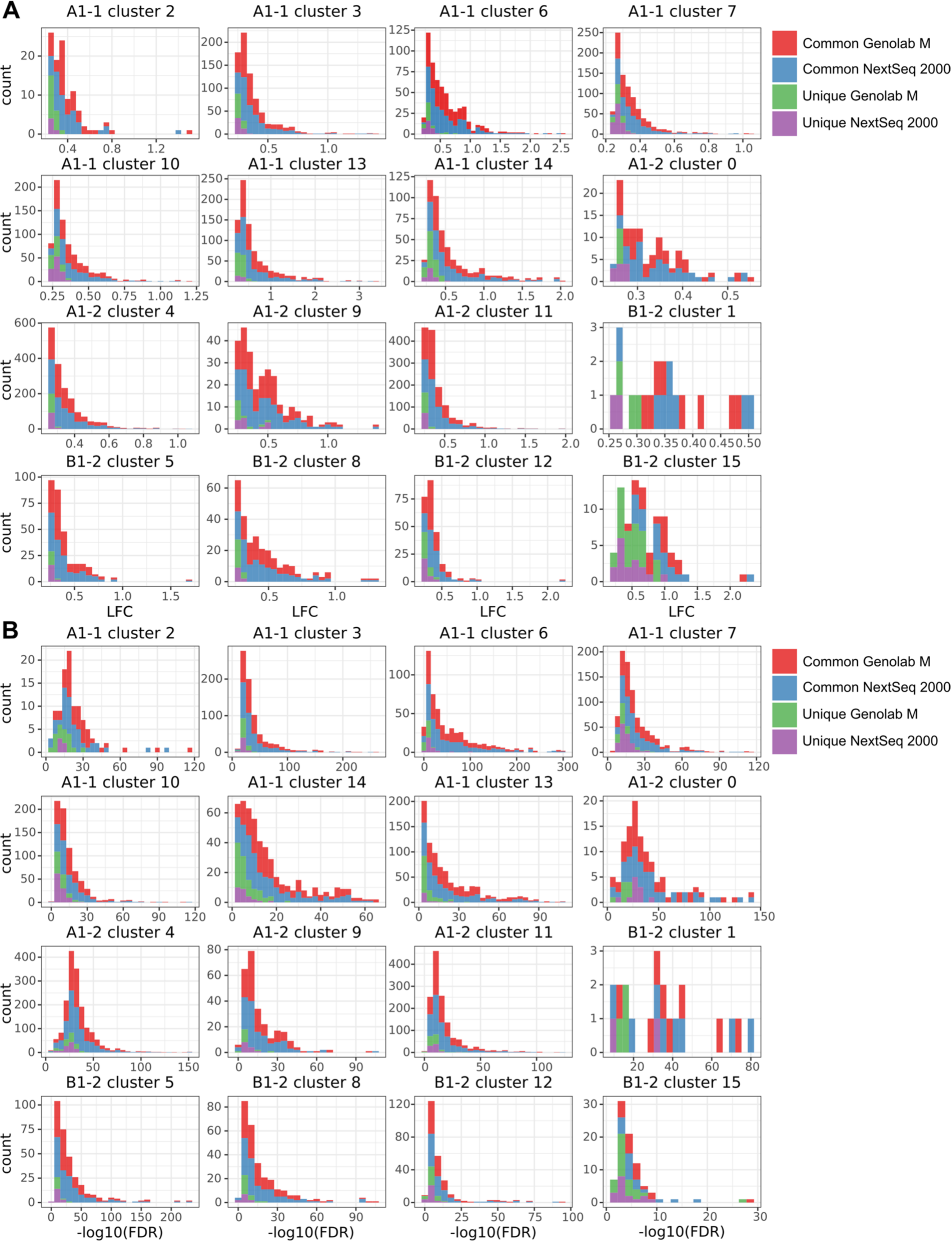
Cluster DEG characterization by the log twofold-change (LFC) and the FDR. **A** LFC distribution of the common and unique DEGs in the clusters. **B** FDR distribution of the common and unique DEGs in the clusters. Count is a number of DEGs

The unique and common DEGs were further characterized to evaluate origin of the platform-specific DEGs. The distribution of DEG counts in tissue spot clusters was visualized to evaluate platform-specific DEGs. Supplementary Figure S2 demonstrates that both unique and common genes had corresponding count coverage; therefore, platform-specific DEGs did not arise from low expressed genes. The distributions of LFC and FDR of DEGs were visualized following count coverage characterization of DEGs. Figure 3A demonstrates distribution of DEG LFC in tissue spot clusters. Predominantly, all platform-specific DEGs had LFC distributed on the edge of the chosen threshold. Furthermore, FDR of platform-specific DEGs (Fig. 3B) tended to have least significant values. The LFC and FDR distributions indicated that platform-specific DEGs predominantly had lowest –log (FDR) and LFC close to the chosen LFC threshold. Therefore, unique DEGs could be primarily arose due to stochastically exceeding in chosen thresholds in both platforms. On the contrary, common DEGs had steadily higher -log(FDR) and LFC compared to unique DEGs. Supplementary Figure S3 demonstrates distinct differences in FDR and LFC between common and unique DEGs detected in clusters of sequenced samples. Top DEGs almost fully consist of overlapping DEGs.

## Discussion

This is the first study demonstrating the use of GeneMind Genolab M platform for 10x Genomics Visium spatial transcriptomics. Moreover, this study first compared sequencing performance of GeneMind Genolab M with Illumina NextSeq 2000 platform. Both platforms employ sequencing by synthesis using a reversible terminator approach [14]. Fluorescently labeled nucleotides are incorporated at a time by polymerase to extending sequencing primer each sequencing cycle. Differences between Genolab M and NextSeq 2000 sequencing techniques include distinct fluorescent labeling approaches. Genolab M utilizes four-color fluorescent labeling marking each nucleotide with distinct fluorescent labels. NextSeq 2000 employs two fluorescent dyes in four combinations: one label for C, another label for T, both labels for A, and no label for G. More specified protocol for GenoLab M sequencing is described in the study by Liu et al. [12].

Our study showed that GeneMind Genolab M sequencing platform generates consistent with Illumina NextSeq 2000 sequencing results. Sequencing quality metrics indicated highly comparable base calling accuracy in UMI sequence, spatial barcode sequence, and probe read sequence. Comparable base calling accuracy resulted in consistent quality control metrics of the raw read mapping and counting. Furthermore, Genolab M and NextSeq 2000 platforms had similar levels of RNA molecule (UMI) and gene detection. Both platforms had identical GC-content profiles of raw reads. Almost all detected genes were overlapped between two platforms; moreover, there were strong correlations between these overlapping genes in spots. Normalization, dimension reduction and clusterization showed particularly consistent results. Identical spot clusters were identified in each sample sequenced using both platforms. Spatial distribution of spot clusters was predominantly similar without obvious differences. The concordance of both platforms was also high in the DEG analysis between obtained clusters. Furthermore, platform-specific DEGs predominantly had lowest FDR and LFC; therefore, unique DEGs could be primarily arose stochastically. Meanwhile, common DEGs detected in clusters of sequenced samples have the highest –log (FDR) and LFC and almost fully consist of top DEGs. Since top DEGs are almost fully overlapped between both platforms, downstream analysis, e.g. gene set enrichment analysis, should produce highly consistent results. All above-mentioned data indicate high concordance between GeneMind Genolab M and Illumina NextSeq 2000 platforms for 10x Genomics Visium spatial sequencing.

## Conclusions

In summary, this is the first study demonstrating that GeneMind Genolab M instrument has similar to Illumina NextSeq 2000 sequencing efficacy and is suitable for 10 × Genomics Visium spatial transcriptomics.

## Materials and methods

### Sample description and library preparation

Three tumor FFPE samples obtained from patients with high-grade serous ovarian cancer were analyzed. Patients were treated in the Department of Gynecological Oncology, Cancer Research Institute of Tomsk National Research Medical Center (Tomsk, Russia). The study was carried out according to Declaration of Helsinki (from 1964, revised in 1975 and 1983) and was approved by the local committee of Medical Ethics of Tomsk Cancer Research Institute; all patients signed informed consent for the study. Shortly, FFPE tissue sections were placed on the Capture Areas of 10x Genomics Visium slides, deparaffinized, and stained by hematoxylin and eosin. Tumor tissue slides were scanned using the Leica Aperio AT2 station (Leica, Germany) and images were processed with Aperio ScanScope. Libraries were prepared using standard protocol provided by 10x Genomics (User Guide | CG000407).

### Sequencing

The obtained cDNA libraries were analyzed following 10x Genomics sequencing recommendation for FFPE Visium libraries. Sequencing cycles comprised 28 bp (Read 1), 10 bp (Index 1 and 2) and 50 bp (Read 2). Illumina NextSeq 2000 sequencing was performed using NextSeq 1000/2000 P2 reagents (100 cycles), whereas GeneMind Genolab M used FCM reagents (150 cycles).

### Bioinformatics processing

Raw sequencing reads were primary processed in the Space Ranger v.1.3 using the count command with default parameters. Raw reads were additionally assessed with FastQC [15] algorithm, and results were visualized with MultiQC [16] tool. After read mapping and counting, resulted feature-barcode matrix was uploaded into R environment and processed via the Seurat [17] R package. The Pearson correlation coefficient was used to evaluate consistence in total UMI and detected gene number between two platforms in tissue spots. Overlapping of the genes between two platforms was visualized using Venn diagram. Normalization of the raw read counts in feature-barcode matrix was performed with the SCTransform [18] function in the Seurat package. The Pearson correlation coefficient between normalized expression profiles of overlapped genes was calculated for each tissue spot in the samples. The SCTransform normalized counts were merged and renormalized without application of batch effect correction. Linear dimension reduction via principal component analysis (PCA) and non-linear dimension reduction via uniform manifold approximation and projection [19] (UMAP) were applied to the merged normalized counts. Clusterization of tissue spots was performed with shared nearest neighbor (SNN) clusterization using first 30 principal components. FindAllMarkers function in the Seurat package was applied to SCT normalized counts using the Wilcoxon Rank Sum test to reveal differentially expressed genes (DEGs) in spatial clusters for each sample separately. FDR correction was performed using the Benjamini–Hochberg method. The distributions of log twofold-change (LFC) and false discovery rate (FDR) of DEGs were used to assess non-overlapping DEGs. The ggplot2 [20] R package was used to visualize most of comparison results.

## Supplementary Information

**Additional file 1: Supplementary Table S1. **FastQC report combined and retrieved via MultiQC.

**Additional file 2: Figure S1.** **A**. Overlap of detected genes. **B**, **C**. Raw read count distribution of the common and unique genes. Count is a number of genes. **Figure S2.** Raw read count distribution of the common and unique DEGs in the clusters. Y-axis is a number of DEGs. **Figure S3.** Box plots describing difference in –log (FDR) and LFC between overlapping (Common) and platform-unique (Unique) DEGs.

## Data Availability

The data underlying this article are available in the article and in its online supplementary material. Raw data are available in NCBI BioProject repository via accession number PRJNA894440. The script was submitted to Github and can be found through the following link: https://github.com/piams/Comparison-of-the-NextSeq-2000-and-Genolab-M-for-spatial-transcriptomics.
