# Supplementary material for: Comparison of the Illumina NextSeq 2000 and GeneMind Genolab M sequencing platforms for spatial transcriptomics
Source: BMC Genomics. 2023 Mar 7;24:102. doi: 10.1186/s12864-023-09192-w (PMC9990361; doi:10.1186/s12864-023-09192-w)
Supplement: Supplementary file 1 — Additional file 1: Supplementary Table S1. FastQC report combined and retrieved via MultiQC. [file 12864_2023_9192_MOESM1_ESM.docx]

Supplementary Table S1. FastQC report combined and retrieved via MultiQC

| Sample Name | Platform | Dups, % | GC, % | Read Length | M Seqs |
| --- | --- | --- | --- | --- | --- |
| A1-1_S1_L001_R1_001 | NextSeq 2000 | 24.90% | 46% | 28 bp | 57.2 |
| A1-1_S1_L001_R1_001 | Genolab M | 9.80% | 46% | 28 bp | 53.6 |
| A1-2_S5_L001_R1_001 | NextSeq 2000 | 65.40% | 47% | 28 bp | 70.4 |
| A1-2_S5_L001_R1_001 | Genolab M | 56.70% | 46% | 28 bp | 64.2 |
| B1-2_S6_L001_R1_001 | NextSeq 2000 | 41.90% | 46% | 28 bp | 88.3 |
| B1-2_S6_L001_R1_001 | Genolab M | 26.20% | 46% | 28 bp | 68.5 |
| A1-1_S1_L001_R2_001 | NextSeq 2000 | 98.00% | 53% | 50 bp | 57.2 |
| A1-1_S1_L001_R2_001 | Genolab M | 95.00% | 52% | 50 bp | 53.6 |
| A1-2_S5_L001_R2_001 | NextSeq 2000 | 94.00% | 53% | 50 bp | 70.4 |
| A1-2_S5_L001_R2_001 | Genolab M | 88.70% | 53% | 50 bp | 64.2 |
| B1-2_S6_L001_R2_001 | NextSeq 2000 | 94.90% | 52% | 50 bp | 88.3 |
| B1-2_S6_L001_R2_001 | Genolab M | 89.00% | 52% | 50 bp | 68.5 |
