# Supplementary material for: Comparison of the Illumina NextSeq 2000 and GeneMind Genolab M sequencing platforms for spatial transcriptomics
Source: BMC Genomics. 2023 Mar 7;24:102. doi: 10.1186/s12864-023-09192-w (PMC9990361; doi:10.1186/s12864-023-09192-w)
Supplement: Supplementary file 2 — Additional file 2: Figure S1. A. Overlap of detected genes. B, C. Raw read count distribution of the common and unique genes. Count is a number of genes. Figure S2. Raw read count distribution of the common and unique DEGs in the clusters. Y-axis is a number of DEGs. Figure S3. Box plots describing difference in –log (FDR) and LFC between overlapping (Common) and platform-unique (Unique) DEGs. [file 12864_2023_9192_MOESM2_ESM.docx]

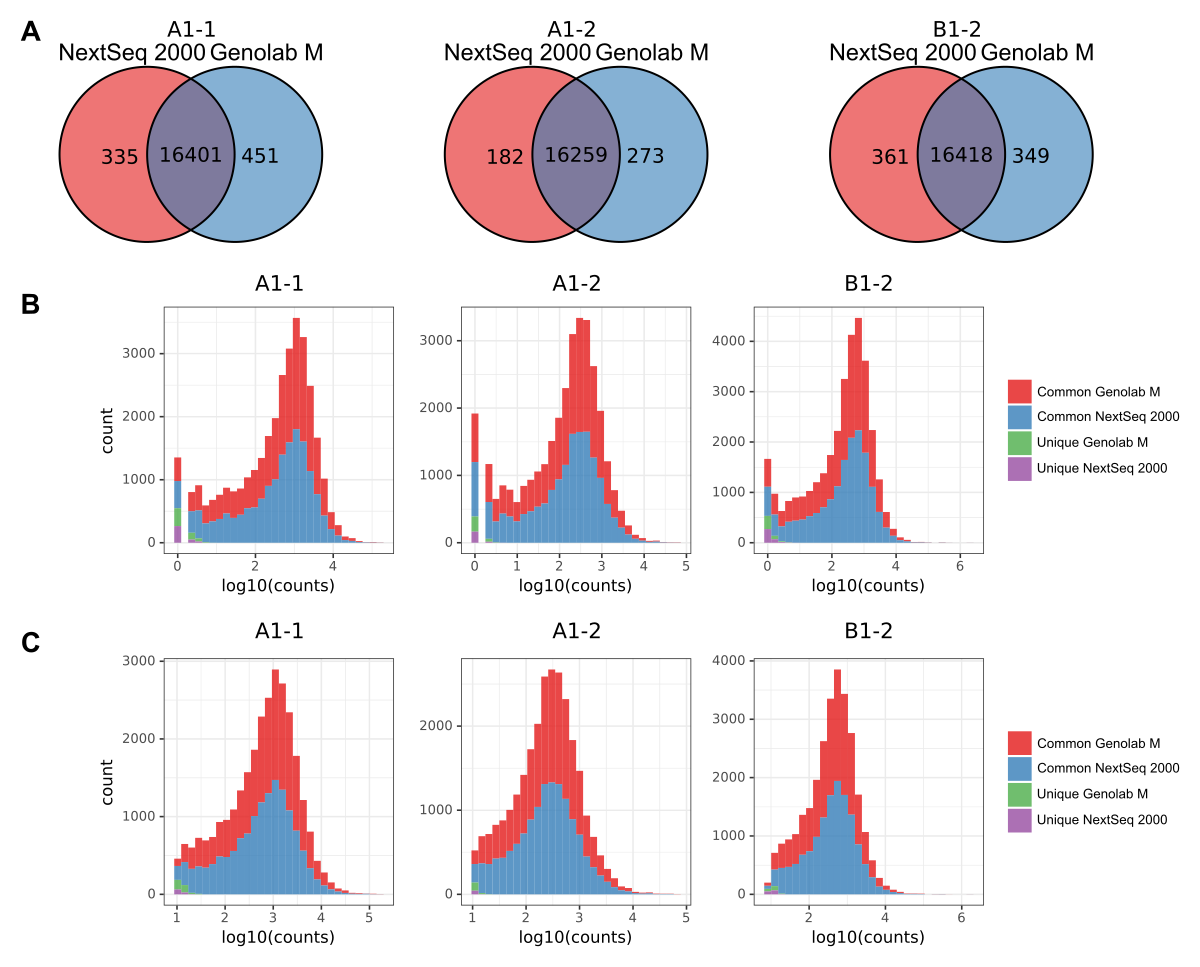


Figure S1. **A**. Overlap of detected genes. **B, C.** Raw read count distribution of the common and unique genes. Count is a number of genes.


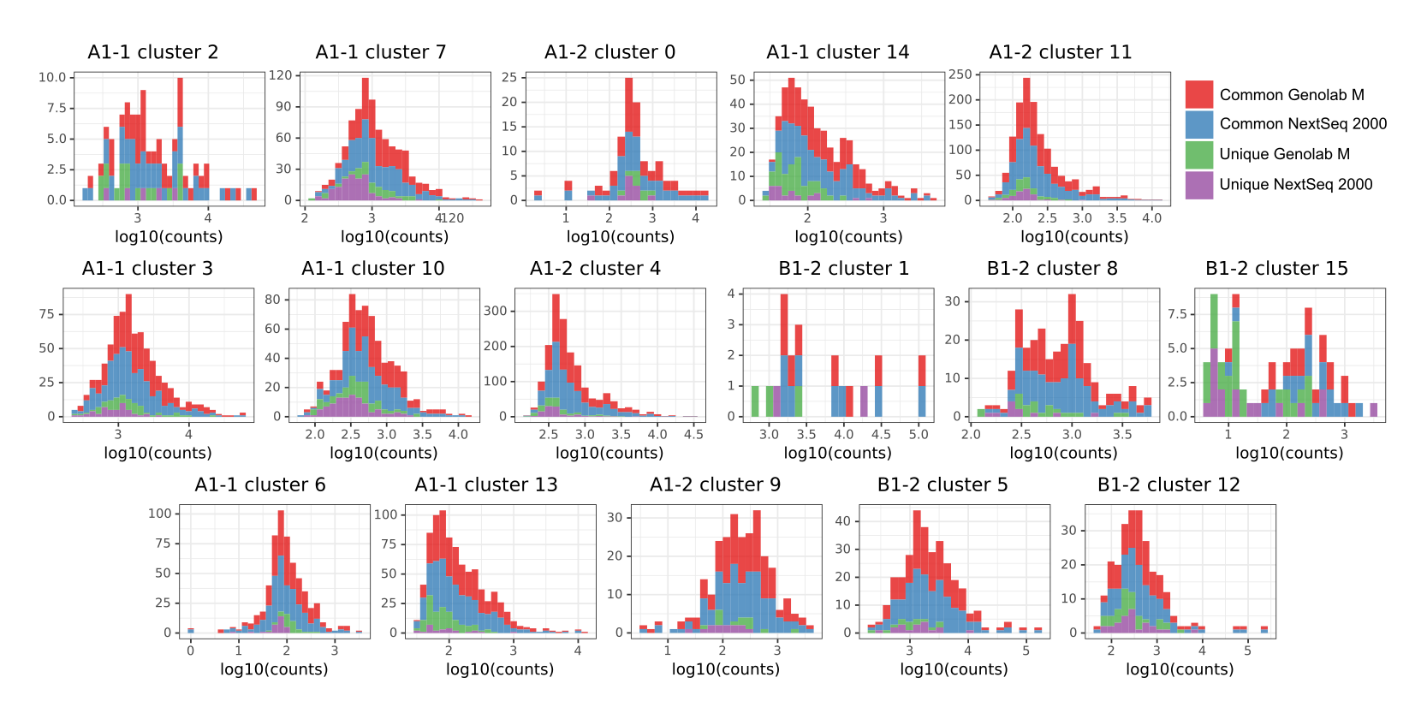


Figure S2. Raw read count distribution of the common and unique DEGs in the clusters. Y-axis is a number of DEGs.


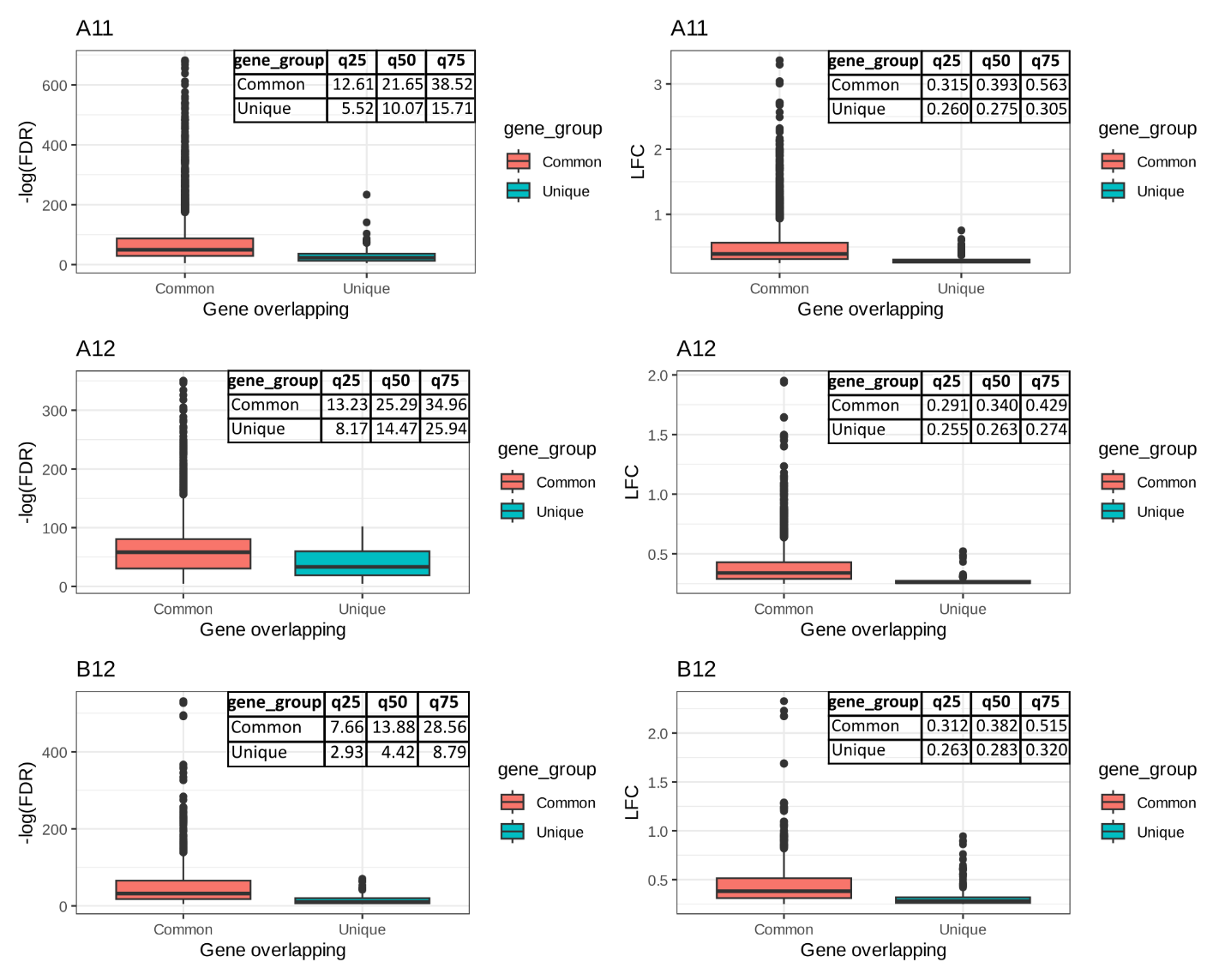


Figure S3. Boxplots describing difference in -log(FDR) and LFC between overlapping (Common) and platform unique (Unique) DEGs.
